# Decentralizing Health Care: History and Opportunities of Web3

**DOI:** 10.2196/52740

**Published:** 2024-03-27

**Authors:** Aditya Narayan, Kydo Weng, Nirav Shah

**Affiliations:** 1 Clinical Excellence Research Center Palo Alto, CA United States; 2 Computer Science Department Stanford University Stanford, CA United States

**Keywords:** Web3, health care, patient-centric, data ownership, decentralization, interoperability, electronic health record (EHR), privacy, blockchain, digital transformation, digital health care, digital health, patient, patients, technological framework, security and privacy, security, privacy

## Abstract

This paper explores the relationship between the development of the internet and health care, highlighting their parallel growth and mutual influence. It delves into the transition from the early, static days of Web 1.0, akin to siloed physician expertise in health care, to the more interactive and patient-centric era of Web 2.0, which was accompanied by advancements in medical technologies and patient engagement. This paper then focuses on the emerging era of Web3—the decentralized web—which promises a transformative shift in health care, particularly in how patient data are managed, accessed, and used. This shift toward Web3 involves using blockchain technology for decentralized data storage to enhance patient data access, control, privacy, and value. This paper also examines current applications and pilot projects demonstrating Web3’s practical use in health care and discusses key questions and considerations for its successful implementation.

## Introduction

The relationship between the development of the internet and health care is a complex one, marked by parallel growth and mutual influence. As we have witnessed with the recent surge in interest surrounding generative artificial intelligence (AI), merely responding to technological innovations within the health care sector is insufficient; instead, we must encourage proactive readiness for the future. Accordingly, in this piece, we aim to explore the current trajectory of internet technologies alongside health care and emphasize the need for a shared vision of the path forward.

In many ways, the evolution of the internet parallels the iterative changes that have defined health care (Table 1). The internet’s earliest form was dubbed Web 1.0, or the “read-only web,” and was filled with static pages, unidirectional flows of information, and minimal opportunities for engagement. This is not unlike the early days of medicine, in which siloed physician expertise left little space for patients to have a voice in their care. Web 1.0 is exemplified by the early adoption of electronic health records (EHRs), which digitized patient records for easier access, albeit in a static form. Over the past several decades, we have transitioned from Web 1.0 to Web 2.0, which emphasizes bidirectional information flows with user-generated content, social networks, and the democratization of information. In the same way, medicine has evolved in light of advances in medical technologies and a growing recognition of the critical role of patients making decisions about their own care. Web 2.0 is characterized by enhanced interactivity and user-generated content, with platforms such as patient portals adding another layer of patient engagement. Regarding public consumption, social media platforms, for instance, facilitated health communities wherein patients could share experiences and build support networks. Further, this era saw the beginning of digital information dissemination in health care. Web-based health information repositories like WebMD provide patients and health care providers with access to medical information. Thus, as a field, health care has made significant progress. However, in the context of Web 2.0, we remain bound by limited interoperability while various entities benefit from the storage, use, and sale of personal information. We are now on the cusp of a new era of the internet—and potentially a new era of health care—with the advent of Web3.

**Table 1:** Parallels between the evolution of the web and medical practice.

Evolution of the web	Evolution of health care
Web 1.0 Static webpages have minimal opportunities for users to engage with content on the page or with other users. There is a unidirectional flow of information from the web page to the user. It is text-based with limited media.	Early medicine Physician-centric—the early stages of medicine were largely dominated by physicians and their expertise. The flow of information and decision-making were unidirectional, from physician to patient. Additionally, there were fewer roles within health care teams, thereby limiting the division of labor and wraparound service delivery. Expertise is siloed by specialty or organ system, with limited crosstalk. Limited patient engagement—patients had minimal input or agency in their own health care. They were often passive recipients of care. Limited technology—the use of technology in health care was limited or rudimentary, with only foundational electronic health record platforms. Technology primarily focused on direct patient care with little emphasis on data management or communication.
Web 2.0 Web 2.0 introduced a read-write web where users could both consume and contribute information. Social networks—the emergence of social networking platforms allowed users to connect, collaborate, and share information. User-generated content—websites began to allow users to upload, modify, and share content. User-driven applications—the development of more advanced web technologies allowed for user-driven applications and services, like blogs, wikis, and media-sharing platforms.	Medicine in the era of technology Increased patient engagement—this era saw a shift toward patient-centered care, where patients have become more active participants in their health care journey with the ability to access (but not own) their health care data. Integration of technology—novel technologies have become deeply integrated into health care, leading to the development of telemedicine, patient portals, and wearable technologies. Interdisciplinary approach—the practice of medicine has become increasingly interdisciplinary, involving teams of diverse health care professionals working together to provide care.
Web3 Web3 uses machine learning, artificial intelligence, and natural language processing to understand and interpret information. Decentralization—data are stored on decentralized networks of computers rather than controlled by individual entities. User control—it is designed to give end users more control over their own data and web-based interactions. It eliminates the need for intermediaries, enabling secure, peer-to-peer interactions.	The future of medicine Data ownership—patients may have full control and ownership of their health data, which could be securely stored and accessed on decentralized networks, improving privacy and interoperability. Advanced health care technologies—the integration of artificial intelligence, blockchain, large language models, and other advanced tools may lead to novel health care solutions like smart contracts for health insurance, predictive health analytics, precision treatments, and so on. Patient autonomy—patients may have increased agency over their involvement in research and clinical care, reinforcing the shift toward truly patient-centered approaches.

Web3 is also referred to as the decentralized web, or the blockchain web, and represents the next stage of the internet wherein data are stored on decentralized networks of computers rather than by individual, centralized entities. Web3 aims to create a more secure, transparent, and user-owned paradigm built on blockchain technology and peer-to-peer networks which enable users to securely interact with one another without the need for intermediaries. Web3 broadly may be thought of as a “new” internet—in which data and web-based interactions are owned and controlled by the end users.

Sharing of patient data initially collected for clinical care or research between health care institutions and third-party companies where it might be used for commercial gain is common [1]. While being cared for, patients may unknowingly agree to the deidentified sharing of their data with external entities. Explicit permission for such sharing is sometimes not required if the data are deidentified but nonetheless limits patient agency. As health care institutions increasingly recognize the commercial value of such data, they engage in activities to refine and monetize these data through specialized third-party organizations. For example, Truveta, founded by a consortium of health systems, exists to structure and commercialize their data assets on behalf of the health systems [2]. The trend extends to hospitals creating and divesting their own spin-off companies for specific data like genetics and annotated pathology. Moreover, companies like Komodo Health aggregate diverse data sets to develop products and services such as clinical trial planning [3]. These commercial use cases highlight the value of the data itself—and the potential for greater patient agency in deciding how their health care data are used. Web3’s architecture could potentially offer more transparent and secure data management solutions for such uses.

With a move to Web3, we may further shift power to patients from insurers, the government, and health systems. For health care, the advent of Web3 promises a transformative shift in how patient data are managed, accessed, and used. Central to this transformation is the use of blockchain technology, which provides a decentralized framework for data storage. This approach ensures that patient data are not confined to a single repository but are distributed across a network, thereby facilitating comprehensive and global access for patients [4]. Moreover, the incorporation of smart contracts and advanced cryptography systems automates the access process, enabling efficient and timely retrieval of data [5]. Web3 additionally empowers patients with bidirectional control over their data. With Web3 integration, patients might contribute to their health records by uploading data from personal health devices, ensuring a more comprehensive health profile that combines clinical and patient-generated data [6]. Privacy protection is a cornerstone of Web3, achieved through advanced cryptographic methods like encryption and secure multiparty computation [7]. These techniques ensure that patient data remain secure and accessible only to authorized individuals, while the immutable nature of blockchain provides a transparent record of access and modifications, enhancing data security. Additionally, Web3 opens avenues for patients to derive novel value from their health data. With proper consent, anonymized data can be used in medical research, with patients receiving compensation in digital tokens [8]. This not only incentivizes data sharing but also allows patients to actively participate in and influence medical research, aligning it with their health interests and ethical preferences. Lastly, although patient data are anonymized for researchers, patients can still access insights derived specifically from their data. These insights include potential genetic risk profiles and early cancer detection. Thus, Web3’s approach to data management redefines the principles of access, control, privacy, and value, pivoting toward a more patient-centric, collaborative, and secure health care ecosystem.

Emerging examples and pilot projects in the health care sector are demonstrating the practical applications of Web3 technologies. For example, companies like Pfizer are leveraging blockchain technology for enhanced traceability in drug supply chains [9]. In patient data management, platforms such as Patientory (Patientory Inc) are empowering patients to store and manage their health data on blockchain, offering unprecedented control and privacy. Furthermore, health care systems are adopting smart contracts to streamline insurance processes, reducing administrative burdens and increasing transparency [5]. In scientific discovery, the field of decentralized clinical trials is also embracing Web3, with platforms like ClinTex using blockchain for secure data sharing and patient recruitment [10-12]. Moreover, the integration of Web3 in biobanks is revolutionizing the management and security of genetic and health data for study [13]. A notable instance of Web3’s collaborative potential is Vibe Bio, a decentralized autonomous organization that unites patients, doctors, investors, and researchers in the pursuit of cures for rare diseases. Vibe Bio leverages blockchain for transparent and collective decision-making in research and funding, overcoming limitations related to the low sample size available for many rare diseases’ studies [14].

Several health care standards, platforms, and techniques are actively paving the way for the implementation of Web 3.0 in health care. For example, the Solid (Social Linked Data) framework, pioneered by Sir Tim Berners-Lee (creator of the World Wide Web), seeks to facilitate a decentralized web by enabling individuals to store their personal health data in “pods” or personal data stores. This grants them the autonomy to manage and share their data securely and efficiently. This democratization of data management resonates with a vision of an open, collaborative internet [15].

Notably, many early Web3 use cases center largely around generating financial value—for example, buying and selling Bitcoin. In fact, countries such as Estonia, have accelerated the Web 2.0 to Web3 transition with significant public investment and widespread adoption of Web3 within the financial sector [16]. However, we wish to focus our discussion on the technology undergirding these transactions and the unique prospect of individual ownership of data. Here, we seek to raise the promise and potential pitfalls of Web3, alongside the associated implications for health care.

## Potential of Web3

Central to the discussion of Web3 is the value of personal ownership of health care data. In the current health care landscape, there is growing concern regarding the ownership and use of patient data. Typically, these data are collected and managed by health systems, service providers, and health insurers. While primarily intended for clinical purposes, there have been discussions and concerns in the public and academic domains about the potential for these data to be leveraged in ways that extend beyond these original intentions [17]. These concerns underscore the importance of clear data governance policies and highlight the ethical implications of data management in health care. The evolution toward systems like Web3 seeks to address these concerns by offering enhanced data ownership and control to patients, thus potentially mitigating the risks associated with data misuse. The global market for data monetization is projected to reach more than US $15 billion by 2030 [18]. This growth highlights the increasing value and potential revenue from data monetization, though it must be noted that business models and strategies for data monetization vary significantly across industries and companies.

While there are instances where health data may be consolidated and disseminated, potentially for profit, there exists a legal and ethical landscape governing these practices. Ethical and policy guidelines, such as those outlined by the US Centers for Disease Control, mandate the responsible use of such data, ensuring the protection of individual privacy and adherence to confidentiality norms. For example, we might consider the UK’s National Health Service. There, a national data opt-out system is in place for the secondary use of confidential health data for research and planning. However, a study revealed that there is limited public awareness of this opt-out system, with many participants being unaware that their anonymized health data could be used for secondary purposes such as research and health planning by entities outside of the National Health Service, including academic and commercial organizations [19].

In addition to facilitating personalized ownership, Web3 offers the potential for digital transformation and an opportunity to overcome the limitations of Web 2.0. Perhaps the most appealing target lies in the reform of EHRs—originally a Web 1.0–based health care technology. Imagine a future in which any patient can view their health records on their cell phone that is hosted by a decentralized network that only they can access. They open the app and it not only shows all their records but also all the scientific papers they contributed to and the insights those papers have generated. Lastly, they can easily change the sharing access of different data points on the device itself. This level of engagement will not just change the relationship between the patient and the health care system, but also the general public with science and research.

Current trends in EMRs already hint at the transition toward a decentralized web as they are evolving to become more patient-focused, with enhancements in data interoperability and security. These developments suggest a shift toward a model where patients play a more active role in managing their health care data. In the foreseeable future, EMRs are likely to further align with decentralized web concepts. This includes the adoption of blockchain technology for secure and transparent data management to streamline health care procedures and empower patient consent. Additionally, the incorporation of decentralized identity solutions in EMRs will enable secure and independent verification of patient identities, reducing dependency on centralized systems. These advancements, which are already being piloted, represent a significant step toward the next generation of web technologies in health care [20]. In contrast to traditionally siloed patient data, Web3 offers the potential of reimagining EHRs as mutable, patient-centric, and patient-owned, where linked data will allow tailored treatments that account for a patient’s unique health history, genetics, environment, and lifestyle. This collective potential may be too great to ignore.

## Five Key Questions to Answer

Despite its theoretical benefits, at present the decentralized web is nascent, and the benefits are largely unrealized. Before Web3 reaches widespread adoption, we believe health care leaders should consider several key questions.

### How Can We Secure Privacy in a Web3 Context?

While Web3 technologies enable ownership, without privacy protections for information sharing, ownership can only go so far. Even with the enhanced security features of Web3 technologies, they are not immune to vulnerabilities. The decentralized nature of blockchain, while reducing certain types of security risks, can also introduce new vulnerabilities. These vulnerabilities may be significant, particularly in light of blockchain’s immutable nature. Researchers have attempted to tackle the challenge of privacy through numerous technical approaches—though many current solutions sacrifice computational speed for privacy [21]. However, emergent solutions have begun to reach the stage of adoption [22-24]. For example, companies such as Onai achieve privacy protection through cryptographic techniques such as secure multiparty computation [25]. Broadly, compliance with legal standards like HIPAA (Health Insurance Portability and Accountability Act) and General Data Protection Regulation is also a must, necessitating regular updates to the governance framework in response to evolving legal requirements. An example is the need to consider data fidelity in the context of General Data Protection Regulation’s “right to be forgotten” in which individuals have the right for their personal data to be erased [26]. Additionally, policies should be patient-centric, prioritizing patient needs and privacy, and include guidelines for ethical data use, especially in research.

### How Will We Facilitate Change Management?

The health care field is conservative by design, and Web3-based technologies often mandate a high level of technical knowledge to implement and use. Further, the centralized model of Web 2.0 is largely incapable of operating alongside Web3’s decentralized approach, and so both will exist in parallel for a time. Thus, there will exist challenges to seamless interoperability between blockchain systems and traditional EHRs leading to data siloes. Beyond this, existing legacy systems within health care institutions may not align seamlessly with the advanced capabilities of Web3 technologies, necessitating significant technical expertise and likely infrastructure upgrades. Practical considerations regarding infrastructure include establishing a robust network infrastructure with adequate bandwidth, setting up blockchain nodes with the requisite server capacity and storage solutions, decentralized identity verification systems to securely manage patient identities, and experience design for user-friendly interfaces. Accordingly, change management toward Web3 systems will require a staged approach supported by iterative stages of implementation.

### How Can Web3 Scale While Maintaining an Equity Lens?

As new technologies arise and each offers novel opportunities, we must remain centered on the ethical mandates of medicine. Access to Web3 mandates the presence of new tools and technical expertise which few health systems possess [27]. Thus, as technologies scale, an opportunity lies in creating an incentive schema that reimburses for equitable adoption without limiting the innovation potential. That is, payers, including insurance companies and government health programs, could establish incentive structures that financially reward health care providers for adopting and effectively using Web3 technologies. These incentives might be linked to specific metrics including, but not limited to, the level of data interoperability achieved, the extent of patient data control facilitated, or the efficiency gains in patient care and administrative processes. Similar to the meaningful use criteria established for EMRs, payers could define a set of criteria or benchmarks for Web3 implementation, offering reimbursements or bonuses to providers who meet these standards. This approach would not only accelerate the adoption of Web3 technologies across different health care settings but also ensure that their integration is aligned with improved patient outcomes and system efficiency. Additionally, there is likely to be user resistance due to the complexities of blockchain technology and the digital divide. Patients in underresourced or rural areas, or those who are less tech-savvy, might find it challenging to engage with Web3-based health care systems, potentially exacerbating existing health care disparities.

### How Can We Avoid Wasteful Spending?

The adoption of Web3 in health care has the potential to help address the persistent “grand challenge” of escalating expenditure in US health care. Examples include enabling more personalized treatment plans to reduce wasteful procedures, improving interoperability to enable efficient care delivery, and enhancing fraud prevention with transparent transactions. However, to realize these benefits, the initial adoption of Web3 must prioritize empirical, high-value principles—focusing on efficiency, appropriateness, and patient-centeredness—to avoid generating new expenses in the rollout process. The integration of Web3 into health care systems, akin to any substantial systemic transition, presents a unique opportunity to reevaluate and realign the system with its foundational principles. Web3’s decentralized architecture, characterized by technologies like blockchain, offers distinct advantages that can be harnessed to enhance these principles more effectively than current systems. Most notably, Web3 technologies provide a framework that can shift the focus back to patient-centered care by giving patients greater autonomy and control over their health data. Such a systemic reorientation during the transition to Web3 not only aligns with the ongoing evolution of health care but also ensures that these fundamental principles are more deeply ingrained in the fabric of health care systems. Through this transition, as described previously, it will be important to curtail costs. The scalability and performance of blockchain in processing large data volumes, given limitations in managing high-throughput data, must be assessed [27]. This scalability challenge could lead to slower transaction processing times and increased costs, potentially hindering the widespread adoption of Web3 in large health care systems.

### How Can the Health Care System Support Policy Makers in Preparing for the Arrival of Web3?

As a field, we must acknowledge that policy regulation is unlikely to keep pace with technological innovation. Accordingly, legal clarity and guardrails will be necessary to ensure industry players, health systems, and academic experts collaborate to create shared endpoints that center on consumer protection, data privacy, and improved care. With fast-growing interest in generative AI and the creation of large language models specific to health care, questions of data ownership, use, and incorporation into new Web3-based tools bring new challenges that have yet to be addressed at scale.

## Considerations for Implementation

### Overview

In the process of transitioning to Web3 systems in health care, a phased approach is paramount for effective change management, implementation, and adoption. Integrating Web3 technologies into health care systems begins with a thorough assessment of existing IT infrastructure to identify areas needing upgrades or changes for Web3 compatibility, which is already a prerequisite for any health system addressing cybersecurity and regulatory compliance. Implementation will also require data migration and ensuring seamless integration with existing health care databases and applications, a step crucial for maintaining data continuity and integrity. Further, it will be essential to define clear rules regarding data ownership, explicitly outlining patient rights and access conditions. Incorporating blockchain technology can facilitate granular control, allowing patients to specify access permissions, thereby creating an auditable trail of data access. This initial phase may be followed by defining specific use cases where Web3 can add significant value, such as in patient data management, supply chain transparency, or facilitating research collaborations. These use cases help to focus the direction of Web3 implementation.

For a health system, initially, strategic planning forms the cornerstone of this process. After prioritizing Web3 opportunities, a detailed implementation roadmap with a sufficient budget must be developed. Stakeholder engagement should include technology experts and vendors, health care providers, IT staff, and patients.

Throughout this process, it will be necessary to scaffold rollout with clinical staff training. As Web3 introduces advanced technologies like blockchain and smart contracts, health care providers must undergo specialized training to become adept in these new systems, focusing on a basic technical understanding and practical clinical application. This shift will also lead to changes in clinical workflows; for instance, the processes for accessing and sharing patient data will evolve, necessitating adaptations to new data retrieval and sharing protocols. Health care professionals will need to adapt to more dynamic decision-making processes due to the real time nature of data updates on blockchain platforms. Additionally, the accuracy and accessibility improvements provided by Web3 could boost clinical efficiency but will require new competencies in data management. Crucially, the patient-centric model of Web3, which grants patients greater control over their data, will transform patient-provider dynamics, placing a greater emphasis on shared decision-making and patient engagement.

The challenges of enforcing privacy protection laws in the Web 3.0 era, where patient control over health care data is paramount, may be addressed through a combination of approaches. These include implementing robust encryption, smart contracts, and security protocols to safeguard patient data against unauthorized access, a measure that gains importance given the existence of extant and ongoing challenges with health care data breaches. To monitor such breaches, comprehensive incident response plans by technology platforms may be developed alongside IT leaders. At a policy level, the development of legal frameworks specifically designed for decentralized data management in health care will be central for providing clear guidelines on liability and actions in the event of a data breach. Finally, the launch of the Web3 system should be coupled with ongoing postimplementation support to address any technical issues or concerns, ensuring the long-term effectiveness and efficiency of Web3 technologies in the health care environment.

### Conclusions

Ultimately, the advent of Web3, with its revolutionary capabilities of decentralization, user data ownership, and advanced privacy, presents transformative possibilities for health care. It portends a shift of power from traditional entities like insurers and health systems to patients—granting individuals the agency to make informed decisions regarding their involvement in research and clinical care. Successful integration of Web3 in health care will hinge on a multidisciplinary approach that combines technological innovation with practical, ethical, and regulatory considerations. By focusing on these areas, we can pave the way for a health care ecosystem that is not only more efficient and patient-centric but also adaptable to the evolving digital landscape.

## Abbreviations

AI: artificial intelligence
EHR: electronic health record
HIPAA: Health Insurance Portability and Accountability Act
Solid: Social Linked Data

## References

[1] Hulsen T (2020). Sharing is caring-data sharing initiatives in healthcare. Int J Environ Res Public Health.

[2] Truveta.

[3] Komodo Health: Healthcare Map.

[4] Saeed H, Malik H, Bashir U, Ahmad A, Riaz S, Ilyas M, Bukhari WA, Khan MIA (2022). Blockchain technology in healthcare: a systematic review. PLoS One.

[5] Vargas C, da Silva MM (2023). Case studies about smart contracts in healthcare. Digit Health.

[6] Zan T, Hu Z (2020). Blockchain-based bidirectional transformations for access control and data sharing in EMRs.

[7] Lee S, Kim J, Kwon Y, Kim T, Cho S (2022). Privacy preservation in patient information exchange systems based on blockchain: system design study. J Med Internet Res.

[8] Shah K, Patt D, Mullangi S (2023). Use of tokens to unlock greater data sharing in health care. JAMA.

[9] Uddin M, Salah K, Jayaraman R, Pesic S, Ellahham S (2021). Blockchain for drug traceability: architectures and open challenges. Health Informatics J.

[10] Maslove DM, Klein J, Brohman K, Martin P (2018). Using blockchain technology to manage clinical trials data: a proof-of-concept study. JMIR Med Inform.

[11] Hang L, Chen C, Zhang L, Yang J (2022). Blockchain for applications of clinical trials: taxonomy, challenges, and future directions. IET Commun.

[12] (2020). Clintex whitepaper: new medicine: faster, safer, smarter. ClinTex.

[13] Mamo N, Martin GM, Desira M, Ellul B, Ebejer JP (2020). Dwarna: a blockchain solution for dynamic consent in biobanking. Eur J Hum Genet.

[14] Vibe Bio.

[15] Sambra AV, Mansour E, Hawke S, Zereba M, Greco N, Ghanem A, Zagidulin D, Aboulnaga A, Berners-Lee T (2016). Solid: a platform for decentralized social applications based on linked data. MIT CSAIL and Qatar Computing Research Institute.

[16] Semenzin S, Rozas D, Hassan S (2022). Blockchain-based application at a governmental level: disruption or illusion? The case of Estonia. Policy Soc.

[17] Wade D (2007). Ethics of collecting and using healthcare data. BMJ.

[18] Ofulue J, Benyoucef M (2022). Data monetization: insights from a technology-enabled literature review and research agenda. Manag Rev Q.

[19] Atkin C, Crosby B, Dunn K, Price G, Marston E, Crawford C, O'Hara M, Morgan C, Levermore M, Gallier S, Modhwadia S, Attwood J, Perks S, Denniston AK, Gkoutos G, Dormer R, Rosser A, Ignatowicz A, Fanning H, Sapey E (2021). Perceptions of anonymised data use and awareness of the NHS data opt-out amongst patients, carers and healthcare staff. Res Involv Engagem.

[20] Sun J, Ren L, Wang S, Yao X (2020). A blockchain-based framework for electronic medical records sharing with fine-grained access control. PLoS One.

[21] Geppert T, Deml S, Sturzenegger D, Ebert N (2022). Trusted execution environments: applications and organizational challenges. Front Comput Sci.

[22] Yao AC (1982). Protocols for secure computations.

[23] Choquette-Choo C, Dullerud N, Dziedzic A, Zhang Y, Jha S, Papernot N, Wang X (2021). CaPC learning: confidential and private collaborative learning. ArXiv. Preprint posted online on February 9 2021.

[24] Huang H, Zhu P, Xiao F, Sun X, Huang Q (2020). A blockchain-based scheme for privacy-preserving and secure sharing of medical data. Comput Secur.

[25] Onai.

[26] Tsirintani M, Serifi M, Binioris S (2019). Digital oblivion (the right to be forgotten): a big challenge for the public hospital management in Greece. Stud Health Technol Inform.

[27] Habib G, Sharma S, Ibrahim S, Ahmad I, Qureshi S, Ishfaq M (2022). Blockchain technology: benefits, challenges, applications, and integration of blockchain technology with cloud computing. Future Internet.

